# Optimization of programmed intermittent epidural bolus volume for different concentrations of ropivacaine in labor analgesia: a biased coin up-and-down sequential allocation trial

**DOI:** 10.1186/s12884-022-04912-8

**Published:** 2022-07-25

**Authors:** Xin Ran, Shuzhi Zhou, Kailan Cao, Peng He

**Affiliations:** 1 Department of Anesthesiology of Ya’an People’s Hospital, Ya’an, China; 2 Department of Anesthesiology of The Second People’s Hospital of Yibin, Yibin, China

**Keywords:** Programmed intermittent epidural bolus, Labor analgesia, Optimal effective dose, Ropivacaine, Sufentanil

## Abstract

**Background and objectives:**

To date, programmed intermittent epidural bolus (PIEB) has been widely used in obstetric analgesia, while no optimal PIEB regimen has been proposed. This study aimed to assess effective analgesia in 90% of women (EV90) with different concentrations of ropivacaine (0.075% and 0.1%) combined with 0.5 µg/mL sufentanil, at an interval of 40 min using the biased coin design-up-and-down method (BCD-UDM), and to explore whether there is a difference in EV90 with the increase of ropivacaine concentration.

**Methods:**

In total, 103 primiparous women were assigned to two groups, including group A (*n* = 52) and group B (*n* = 51). Parturients in group A were treated with 0.075% ropivacaine and 0.5 µg/mL sufentanil, while those in group B were treated with 0.1% ropivacaine and 0.5 µg/mL sufentanil. Used the biased coin up-and-down sequential allocation method to determine the EV90. The secondary outcomes were sensory block level, motor block, and adverse events (hypotension, urinary retention, and pruritus).

**Results:**

The results revealed that EV90 was 10 mL (95% confidence interval (CI):8.03–11.54) in group A, and EV90 was 9 mL (95% CI:7.49–10.51) in group B by the isotonic regression method. The highest level of the sensory block was T8, and the lowest was T12. No case of hypotension was recorded,and only 4 parturients complained of motor block.

**Conclusion:**

With an interval of 40 min, the optimal PIEB bolus volume of 0.075% ropivacaine and 0.5 µg/mL sufentanil was 10 mL, 0.1% ropivacaine and 0.5 µg/mL sufentanil was 9 mL. Moreover, the PIEB volume decreased along with the higher concentration of ropivacaine.

**Trial registration:**

ChiCTR registration number: ChiCTR2000040917. Registration date: December 15, 2020.

## Introduction

Neuraxial analgesia is considered the gold standard in labor analgesia, providing the most effective pain relief during childbirth [1]. PIEB was proposed as a more efficacious technique to maintain labor epidural analgesia compared with continuous epidural infusion(CEI). Studies comparing PIEB with CEI have shown that PIEB is associated with the reduced local anesthetic consumption [2–4], a lower incidence of breakthrough pain [2, 4, 5], the reduced incidence of cesarean delivery [6], and a greater maternal satisfaction [2, 4, 6–8]. There is evidence that PIEB regimens decrease motor block and instrumental deliveries [8, 9]. However, the optimal PIEB regimen has still remained to be determined.

The optimal PIEB regimen has varied significantly among different studies [9–15]. Bittencourt et al. [16] used a PIEB volume of 10 mL of bupivacaine 0.0625% with fentanyl 2 µg/mL, in which the interval varied between 30 and 60 min, and found an optimal interval of approximately 40 min. Zhou et al. [17] designed a study to identify the optimal interval for PIEB using 10 mL of ropivacaine 0.08% and sufentanil 0.3 µg/mL; the study found that with a fixed 10 mL dose of ropivacaine 0.08% with sufentanil 0.3 µg/mL, the optimal PIEB interval was about 42 min. Another study yielded similar results [15], suggesting that 40 min may be an optimal PIEB interval.

To date, few studies have concentrated on the optimal PIEB volume. Zakus et al. determined the optimal PIEB volume at a 40 min interval to provide effective analgesia in 90% of women, without the use of patient-controlled epidural analgesia (PCEA), and the volume was in the range of 7—12 mL. This study suggested that the optimal PIEB volume of bupivacaine 0.0625% with fentanyl 2 µg/mL administered at a fixed interval of 40 min was approximately 11 mL [18]. Epstein et al. also demonstrated that 10 mL boluses of bupivacaine 0.0625% with fentanyl 2 µg/mL delivered every 40 min produced an effective analgesia without breakthrough pain in 90% of women [15].

Ropivacaine has increasingly been replaced with bupivacaine in obstetric anesthesia because it causes less motor blockade and damage to cardiovascular system and central nervous system toxicity [19, 20]. However, it could not be assumed that the optimal PIEB volume with ropivacaine and sufentanil was the same as that of PIEB with bupivacaine and fentanyl. It is clinically of great importance to determine the optimal PIEB volume for the mixture of ropivacaine and sufentanil.

The present study aimed to evaluate the EV90 with different concentrations of ropivacaine (0.075% and 0.1%) combined with 0.5 µg/mL sufentanil, at an interval of 40 min using the BCD-UDM, and to explore whether there is a difference in EV90 with the increase of local anesthetic concentration.

## Methods

The present study was approved by the Ethics Committee of Ya’an People’s Hospital (Ya’an, China; Approval No. 202015). Parturients who were admitted to the Ya’an People’s Hospital from March 1, 2021, to November 30, 2021, were enrolled. We obtained written informed consent from all parturients prior to enrollment. The study was conducted in accordance with the Consolidated Standards of Reporting Trials (CONSORT) statement.

Inclusion criteria were primiparous women with American Society of Anaesthesiologists (ASA) class II-III, gestational age between 37 and 42 weeks, singleton pregnancy, regular contractions, and cervical dilation of 2–3 cm. We excluded women who had a contraindication to epidural analgesia, hypersensitivity to ropivacaine or sufentanil, and those who refused to participate in the study. Withdrawal criteria were failure to perform epidural anesthesia, VAS > 3 after loading dose,cesarean section in the first stage of labor, and loss to follow-up. Parturients were assigned into two groups: group A (ropivacaine 0.075% and sufentanil 0.5 µg/mL) and group B (ropivacaine 0.1% and sufentanil 0.5 µg/mL). The recruitment of women in group A was completed first, followed by group B.

After the parturients arrived in the delivery room, maternal heart rate, blood pressure, oxygen saturation, respiration, and fetal heart rate were continuously monitored. The epidural puncture was performed at the L3–4 vertebral interspace by the midline approach. Using the traditional surface landmarks-based approach. Local infiltration was carried out using 3 mL of 2% lidocaine. Using a loss of resistance to saline technique with a 17G puncture needle, a 19G multiport wire-reinforced epidural catheter (AS-E/SII; TuoRen Medical Co., Ltd., Changyuan, China) was inserted into the epidural space with a depth of about 4 cm. All epidural catheter insertions were performed by a consultant. Then, 3 mL of 1.0% lidocaine (Zhaohui Pharmaceutical Co., Ltd., Shanghai, China) was injected to rule out the possibility of subarachnoid injection or intravenous injection in the next 5 min. Subsequently, a loading dose was administered consisting of two 5 mL boluses of ropivacaine (Hengrui Pharmaceutical Co., Ltd., Nanjing, China) with sufentanil (Yichang Renfu Pharmaceutical Co., Ltd., Wuhan, China), given 5 min apart. To continue with the study, we required that the pain score of the visual analog scale (VAS) ≤ 3 was achieved within 20 min of administering the loading dose.

Subsequently, a solution of ropivacaine (0.075% or 0.1%) with sufentanil 0.5 µg/mL was administered via a PIEB pump (ZZB-IV; Apon Co., Ltd., Nanjing, China), and the infusion rate was 200 mL/h. The first bolus was given at 40 min after the completion of the loading dose, and all subsequent PIEB doses were given at a fixed interval of 40 min. Besides, PCEA was set to 5 mL/time, the lock time was 15 min, and the maximum dose per hour was 32 mL. The range of PIEB volume was between 7 and 12 mL. Each woman was explained how to use PCEA for breakthrough pain and instructed to press the PCEA button if she felt uncomfortable. If the woman pressed the PCEA button or asks for a manual bolus, the bolus was considered inadequate.

The first woman enrolled in the study was administered a bolus of 7 mL. The bolus for the subsequent woman was determined by the response of previous woman and the BCD-UDM. If the bolus volume did not provide adequate analgesia, the bolus for the next woman was increased by 1 mL. In case of adequate analgesia, the next woman’s bolus was coin-randomized with an 11% probability of decreasing by 1 mL and an 89% probability remained the same. In case of adequate analgesia at 7 mL or inadequate analgesia at 12 mL, the bolus for the next woman did not change. The BCD-UDM allocation was carried out using a computer-generated list of random responses. A research assistant used this list to provide the PIEB volume setting for the next woman in a sealed envelope. An anesthetist, who was blinded to the objective of the study, set up the epidural infusion pump. The epidural infusion pump was covered to blind the participant, investigator and nurse.

Baseline data of each woman included physical characteristics, type of labor (spontaneous, instrumental,or cesarean delivery), and use of oxytocin. The sensory block level to ice was detected by applying ice in the midclavicular line, VAS score (0–10, where 0 = no pain and 10 = the highest level of pain), and motor block score (modified Bromage score(MBS): 0 = no motor block; 1 = inability to raise extended leg but able to move knees and feet; 2 = inability to raise extended leg and move knee but able to move feet; 3 = complete motor block of limb). All assessments were completed by a blinded investigator at 20 and 40 min after ending the loading dose, followed by every hour thereafter until the completion of the study.

The primary outcome was adequate analgesia, which was defined as no use of PCEA or request for manual boluses until the woman’s cervix was fully dilated. Secondary outcomes included sensory block level, motor block, and adverse events (hypotension that was defined as a decrease in systolic blood pressure by 20% of baseline, urinary retention, and pruritus).

Trials with a BCD-UDM need a sufficient sample size, which may increase statistical accuracy and make the standard errors smaller [21]. Therefore, to estimate the EV90, We appropriately increased the sample size to include 52 cases in group A and 51 in group B. The EV90 was calculated by isotonic regression, and 95% confidence interval (95% CI) was calculated by bootstrapping. Isotonic regression is a well-described variant of restricted least-squares regression that constrains the point estimates to be either monotonically increasing or monotonically decreasing. Isotonic regression has favorable statistical properties. We used the dose estimator μ3. The isotonic estimator μ3 has a smaller biasand MSE, which was defined as the linearly interpolated estimator of the target dose. It was derived from the two consecutive boluses that success rates enclosed the value of probability of effect ‘Г’ [22]. Statistical analysis was performed using R 3.6.2 software. The descriptive summaries of some indicators of secondary outcomes were performed.

## Result

**Fig. 1:**
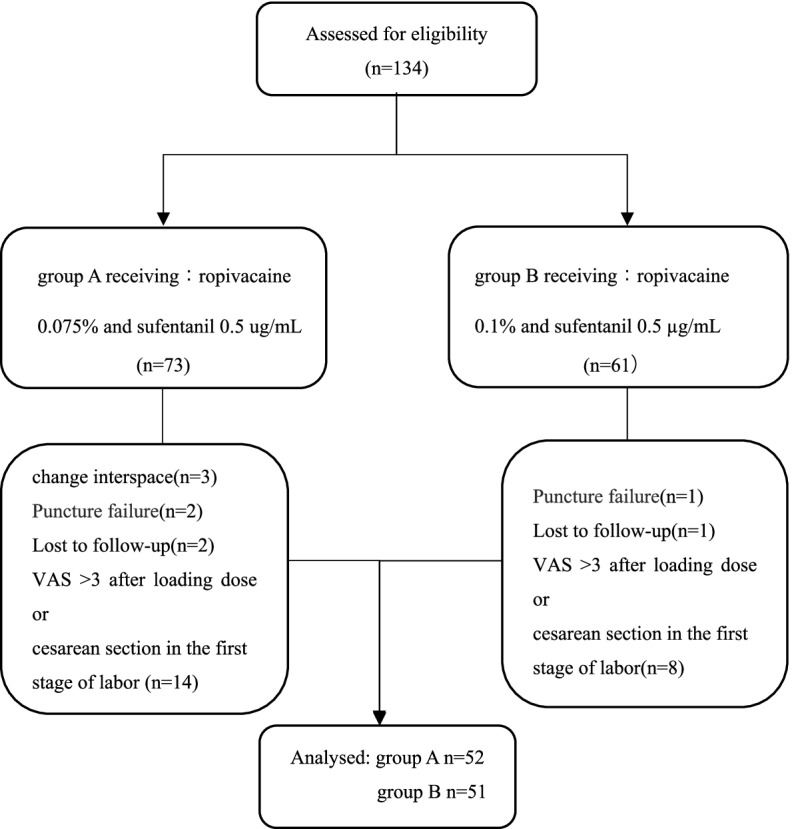
The study flowchart

**Fig. 2:**
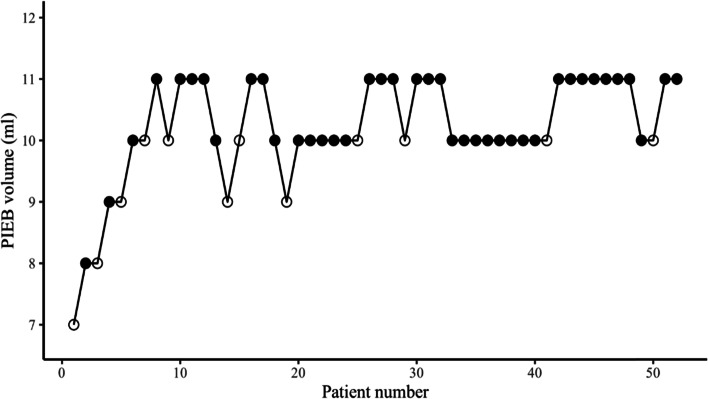
Individual responses of women to different programmed intermittent epidural bolus (PIEB) volumes in group A. Open circle: ineffective PIEB volume; Filled circle: effective PIEB volume

**Fig. 3:**
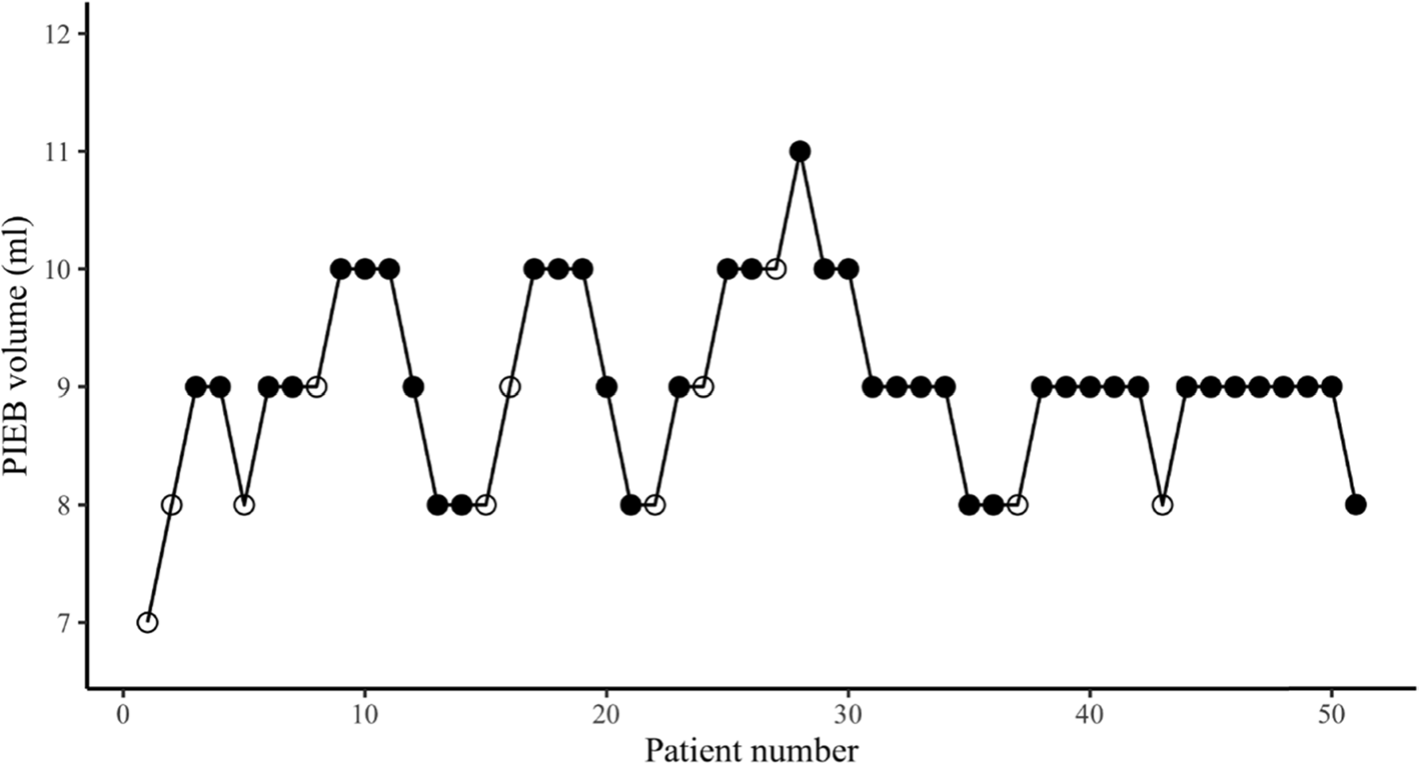
Individual responses of women to different programmed intermittent epidural bolus (PIEB) volumes in group B. Open circle: ineffective PIEB volume; Filled circle: effective PIEB volume

A total of 134 parturients were enrolled in the study. Finally, group A had 52 parturients and group B had 51 parturients included in the data analysis. The study flowchart is shown in Fig. 1. Parturient’s demographic and labor characteristic are summarized in Table 1. The parturients allocation sequences and responses to different PIEB volume are illustrated in Figs. 2 and 3.

**Table1:** Parturients’ characteristics

	Group A(*n* = 52)	Group B(*n* = 51)
age(Y)	26.25 ± 3.34	26.49 ± 2.80
weight(Kg)	67.95 ± 7.89	69.29 ± 9.18
height(m)	1.60 ± 0.04	1.61 ± 0.05
BMI(Kg/m^2^)	26.62 ± 2.90	26.71 ± 2.88
Gestation(weeks)	39.08 ± 0.99	38.98 ± 0.93
Labour (n (%))		
Spontaneous	45(86.5%)	44(86.28%)
instrumental	0(0)	1(1.96%)
cesarean delivery	7(13.5%)	6(11.8%)
Oxytocin administration, n (%)	16(30.8%)	15(29.4%)
Cervical dilation at onset of study(cm), median (IQR)	2(2,3)	2(2,3)
hourly consumption of ropivacaine(mg)	11.25	13.5
Adverse event (n (%))		
pruritus	2(3.85%)	1(1.96%)
hypotension	0(0)	0(0)
urinary retention	0(0)	0(0)

Values are presented as mean (SD), number (%), or median (IQR), *BMI* Body mass index, *IQR* Interquartile range, *SD* Standard deviation, *Y* Years old, *Kg* Kilogram, *m* Meters

The estimated EV90 was 10 mL (95%CI:8.03–11.54) and 9 mL (95%CI:7.49–10.51) with the isotonic regression method in group A and group B, respectively. In 9, 10, and 11 mL groups, effective analgesia was achieved in 25%, 70.83%, and 100% of women in group A, respectively. In 8, 9, and 10 mL groups, effective analgesia was achieved in 50%, 88.46%, and 90.91% of women in group B, respectively. The proportion of women with successful analgesia for each PIEB volume is shown in Table 2. For those patients who did not respond to analgesia, the majority of them received PCEA within 2 h. The details of PCEA timing are summarized in Table 3.

**Table 2:** The effectiveness of each PIEB volume

PIEB bolus volume(mL)	Rate of effective analgesia n(%)	
	Group A(*n* = 52)	group B(*n* = 51)
7	0(0)	0(0)
8	1(50.00%)	6(50.00%)
9	1(25.00%)	23(88.46%)
10	17(70.83)	10(90.91%)
11	21(100.0%)	1(100.00%)
12	0(0)	0(0)

**Table 3:** Time of starting PCEA in both groups

Time to start PCEA and PIEB volume(min, V)		
Patient number	Group A(*n* = 12)	Group B(*n* = 11)
1	55(7)	60(7)
2	62(8)	143(8)
3	74(9)	94(8)
4	104(10)	112(9)
5	141(10)	67(8)
6	93(9)	107(9)
7	150(10)	97(8)
8	100(9)	70(9)
9	156(10)	55(10)
10	115(10)	110(8)
11	76(10)	135(8)
12	92(10)	

The highest level of sensory block in the included women was T8, and the lowest was T12.No sensory block higher than T6 was observed. No parturient had motor block with a MBS score greater than 1 in both groups. Women who received 10 mL PIEB volume and above exhibited a trend toward higher maximum sensory block levels. The results are summarized in Table 4.

**Table 4:** Sensory block, motor block incidence for different PIEB volume

	Volume of PIEB(mL)											
	7		8		9		10		11		12	
	Group A (1)	Group B (1)	Group A (2)	Group B(12)	Group A(4)	Group B(26)	Group A(24)	Group B(11)	Group A(21)	Group B(1)	Group A(0)	Group B (0)
highest Sensory block												
T7	0	0	0	0	0	0	0	0	0	0	0	0
T8	0	0	0	0	0	2	3	4	13	1	0	0
T9	0	0	0	0	1	10	8	5	8	0	0	0
T10	0	0	1	6	1	11	9	1	0	0	0	0
T11	0	1	0	1	2	1	4	1	0	0	0	0
T12	1	0	1	5	0	2	0	0	0	0	0	0
Modified Bromage score												
0	1	1	2	12	4	25	23	10	20	1	0	0
1	0	0	0	0	0	1	1	1	1	0	0	0
2	0	0	0	0	0	0	0	0	0	0	0	0
3	0	0	0	0	0	0	0	0	0	0	0	0

Only one parturient in group B and 2 parturient in group A had pruritus. No maternal hypotension or urinary retention were recorded (Table 1).

## Discussion

In the present study, the results demonstrated that 10 mL was the optimal PIEB volume for 0.075% ropivacaine, and 9 mL was the optimal PIEB volume for 0.1% ropivacaine. The corresponding hourly consumption of ropivacaine was 11.25 and 13.5 mg/h in group A and group B, respectively. The incidence of motor block and adverse effects was very low, which was similar to other studies [16, 17, 23]. Although three parturients complained of pruritus, no case of hypotension or urinary retention was reported.

Wu et al. found that 10 mL PIEB volume was better than 8 and 5 mL with 0.1% ropivacaine combined with 0.33 ug/mL sufentanil [24]. Similar results were also observed in other studies [13, 23]. The results suggested an approximately 10 mL PIEB volume, maybe a better setting, regardless of the different concentrations of anesthetics.

Our study found that when the PIEB volume was < 10 mL in group A or < 9 mL in group B, the effectiveness of analgesia was significantly reduced in the two groups, suggesting that it is not possible to reduce the PIEB volume below EV90 with our current PIEB regimen without compromising the quality of analgesia. Similar results were also reported previously [15].

Moreover, in our PIEB regimen at a fixed interval of 40 min, the EV90 showed a decreasing trend with the increase of ropivacaine concentration. But one point of interest was that the hourly consumption of ropivacaine was comparable between the two different anesthetic recipes. Ricardo et al. found that when the concentration of bupivacaine increased from 0.0625% to 0.125% at a fixed bolus volume of 10 mL, the optimal interval was shortened from 40 to 35 min [16]. This suggests that increasing local anesthetic concentration can shorten the optimal interval and reduce the bolus volume, while the increase of local anesthetic concentration may be associated with more complications, such as motor block and the upper sensory block level.

The highest level of sensory block in the present study was T8, which was lower than previously reported levels [16, 18, 25, 26]. In general, the highest level of sensory block is associated with the puncture position, anesthetic and its concentration, pump speed, and other factors. In our study, the puncture position was L3-L4, catheter was inserted by 3 cm, the speed of PIEB pump was 200 mL/h, and the maximum tested PIEB volume was 11 mL, which were all lower or smaller than their corresponding variables used in previous reports. This may explain why the sensory block level was lower in our study.

There are some limitations in the present study. First, the study was conducted under a fixed PIEB interval and PIEB pump speed, hindering the generalization of the results. These limitations made the conclusions only suitable for these strictly set conditions. Second, as we only followed up women during the first stage of labor, we do not know how well our regimen works during the second stage of labor. Detailed study for different labor stages may find better PIEB regimen for labor analgesia.

In conclusion, the EV90 of 0.075% ropivacaine and 0.5% sufentanil with a fixed interval of 40 min is 10 mL, and 9 mL for 0.1% ropivacaine and 0.5% sufentanil. The PIEB volume decreased along with the higher concentration of ropivacaine. However, there was no significant difference in the local anesthetic hourly consumption between the two groups. The incidence of motor block and adverse effects was very low in the two groups. Our results suggested that approximately 10 mL PIEB volume may be the optimal PIEB regimen, regardless of the different concentrations of anesthetics. Further attention should be paid to the ropivacaine concentration, different local anesthetics, and the optimal bolus interval to optimize the PIEB regimen. Moreover, more research is needed in the future to optimize the PIEB program and to address the analgesic needs of parturients in all stages of labor.

## Data Availability

All data generated or analysed during this study are included in this published article.

## Abbreviations

PIEB: Programmed intermittent epidural bolus
BCD-UDM: Biased coin design up-and-down sequence method
EV90: Optimal effective bolus volume required for effective analgesia in 90% of women
95% CI: 95% Confidence Interval
CEI: Continuous epidural infusions
VAS: Visual analogue scale
MBS: Modified Bromage score
PCEA: Patient-controlled epidural analgesia
